# Extraocular Muscle Transplantation Surgery for Primary Treatment of Extra Large-angle Squint

**DOI:** 10.18502/jovr.v19i2.12791

**Published:** 2024-06-21

**Authors:** Adedayo O. Adio, Chinyelu N. Ezisi, Elizabeth D. Nkanga

**Affiliations:** ^1^Pediatric Eye Clinic, Department of Ophthalmology, University of Port Harcourt Teaching Hospital Port Harcourt, Rivers State, Nigeria; ^2^Pediatric Eye Clinic, Alex Ekwueme Federal University Teaching Hospital, Abakaliki, Ebonyi State, Nigeria; ^3^Calabar Children’s Eye Centre, Department of Ophthalmology, University of Calabar Teaching Hospital Calabar, Cross River State, Nigeria; ^5^Adedayo O. Adio: https://orcid.org/0000-0002-9196-5187

**Keywords:** Expanded Surgical Table, Muscle Transplantation, Squint

## Abstract

**Purpose:**

Large-angle horizontal ocular deviations will commonly require bilateral surgery to correct the primary ocular deviation. However, considering the need for full correction with one surgical procedure and patients' reluctance to be operated on the good eye, such large horizontal ocular deviations may be managed with true muscle transplantation. The authors present a case series of patients who underwent this procedure and develop a surgical table to guide management.

**Methods:**

Patients with horizontal squints measuring 80 prism diopters (PD) or larger in all age groups who underwent extraocular muscle transplantation surgery between January 2019 and June 2022 in Nigeria were included. Preoperative deviation of the squint, sensory evaluation, surgical dosage, and outcomes were documented. Part of the resected muscle was transplanted to give additional recession in the antagonist muscle. Success was defined as deviation corrected by 60% or more or postoperative ocular alignment within 10 PD or less, six weeks postoperatively.

**Results:**

Fourteen patients with extra-large-angle strabismus were operated. Male/female ratio was 0.6:1. Mean preoperative deviation of 89.6 ± 9.3 collapsed to 6.6 ± 1.8 PD at six weeks and continued to improve to a mean deviation of 2.5 PD at six months postoperatively. When the subgroup of patients who were < 18 years were analyzed, the outcome was equally successful; preoperative deviation of 89.4 PD collapsed to 1.4 PD, six months postoperatively. There were equal success rates when those with sensory strabismus were compared with those with binocular vision; preoperative deviation of 92.5 PD in the sensory group and 88.5 PD in the binocular group collapsed to 5.9 PD and 1 PD, respectively, six months after surgery.

**Conclusion:**

A viable alternative for treating extra-large-angle strabismus in adults and children in developing countries was described with good postoperative outcome. In addition, a new expanded surgical dosage table for muscle transplantation surgery corrections of up to 130 PD was developed.

##  INTRODUCTION 

Surgical management of extra-large-angle horizontal ocular deviations poses a huge challenge to the strabismologists whose goal is to achieve optimal ocular alignment and thus prevent complications that can lead to visual impairment.^[1]^ This requires surgery on the three or four horizontal recti muscles; therefore, performing surgery in the two eyes.^[2,3,4,5]^ However, correction of these extra-large-angle horizontal deviations can be sub-optimal despite giving the highest possible corrections using available surgical tables.^[2,3,4,5]^

Standard surgical correction doses developed by Kenneth Wright,^[6]^ stated sizes in millimeters of horizontal rectus correction for deviations up to 70 prism diopters (PD). Von Noorden,^[7]^ Rosenbaum,^[8]^ Stallard,^[9]^ and Kushner^[10]^ had no documented corrections for > 70 PD deviations, thus creating the need to develop surgical operation doses for extra-large-angle deviations.

Late presentation is a major challenge among squint patients in Africa.^[11]^ This delay causes ocular deviations to develop into extra-large angles. Another challenge is loss to follow-up amongst patients that accept surgical correction. The strabismologist is thus faced with the challenge of achieving optimal correction in one surgical session. This is sometimes unable to fully correct these large-angle ocular deviations, with the available recommended surgical doses. In addition, in cases of sensory exotropia (XT) or esotropia (ET), the patient may be willing to undergo surgery only in the eye with poor vision.

Surgical correction of the four horizontal muscles in one session, may predispose the patient to a longer morbidity period, prolonged surgery with increased risk of infection, the prolonged effect of anesthesia, and the risk of under-corrections, creating a need for repeated surgeries. There is also the risk of duction limitations and inducing lateral incomitance.^[12,13]^ Large recessions may also lead to the widening of the palpebral fissure while large resections may lead to the narrowing of the palpebral fissure, and these are not desirable.

Muscle transplantation therefore can be a viable surgical treatment option to mitigate these challenges and achieve optimal alignment with minimal complications in just one surgical session. Muscle transplantation is a surgical procedure, which involves suturing a resected autograft of the rectus muscle (e.g., medial rectus [MR] muscle) to the disinserted end of the opposite rectus muscle (e.g., lateral rectus [LR] muscle) thereby lengthening it and further recessing this elongated rectus muscle to correct extra-large-angle horizontal deviations.^[14]^ Optimal correction can be achieved by working on two recti muscles in one eye using this technique; thus reducing surgical time and anesthesia time while leaving the other eye in its virgin state.

Extraocular muscle transplantation is safe^[15]^ and is recommended when a patient is reluctant to have surgery on the preferred eye, in the event of large deviations (e.g., heavy eye syndrome)^[16,17,18]^ if the forced duction test shows a tight MR (e.g., long-standing ET) and can be done in combination with loop myopexy.^[19]^ It can also be carried out in both eyes with good results where indicated.^[1]^

To the best of our knowledge, no study on muscle transplantation for the treatment of squints has been published in Nigeria and West Africa at the time of this report. This paper therefore discusses the outcome of a case series of muscle transplantation surgeries done in Southern Nigeria and presents the preliminary outcomes.

##  METHODS

Cases of muscle transplantation surgery for treatment of squints carried out between June 2019 and June 2022 in the teaching hospitals affiliated to the University of Port Harcourt, University of Calabar, and Alex Ekwueme Federal University, performed by three surgeons with complete data up to six months postoperatively were included in this descriptive study. Any squint up to 70 PD is considered large. Extra-large-angle squint was therefore defined as any squint equal to or larger than 70 PD. Pre-existing standard surgical tables for muscle transplantation surgeries were used to determine the dose of surgical correction to administer. However, this table did not extend beyond correction for 90 PD; thus corrections greater than this value were further developed by the current authors.^[20]^ Exclusion criteria were previous squint surgery and previous cataract or glaucoma surgery.

**Table 1:** All patients who had extraocular muscle transplantation surgery carried out for extra-large strabismus in Nigeria between 2019 and 2021


**Patient**	**Age (yr)**	**Gender**	**Log MAR**	**Spherical equivalent**	**Stereopsis (Secs. of arc)**	**Type of squint**	**Secondary ocular diagnosis**	**Pre-op squint value-distance/ near (PD)**	**Muscle transplanted**	**Deviation 6 weeks Post-op (PD)**	**Size of deviation 6 months post-op (PD)**
Patient 1	24	F	0.0	0	400	Exotropia	Myopia	70/70	Medial Rectus	8	4
Patient 2	2	F	0.6	+0.75	< 500	Exotropia	Nil	90/90	Medial Rectus	Ortho	Ortho
Patient 3	5	M	0.2	0	400	Exotropia	Nil	90/90	Medial Rectus	20	2
Patient 4	24	F	0.0	+1	< 500	Sensory Exotropia	Corneal opacity	80	Medial Rectus	15	15
Patient 5	35	M	0.0	0	< 500	Exotropia	Nil	90/90	Medial Rectus	10	Ortho
Patient 6	25	M	0.0	-4	< 500	Exotropia	Myopia	90/90	Medial Rectus	4	4
Patient 7	39	F	0.0	0	< 500	Sensory Exotropia	Optic atrophy	110	Medial Rectus	Ortho	Ortho
Patient 8	1	F	0.6	0	< 500	Esotropia	Nil	90/90	Lateral Rectus	Ortho	Ortho
Patient 9	22	F	0.0	0	< 500	Exotropia	Nil	100/95	Medial Rectus	Ortho	Ortho
Patient 10	5	M	0.0	0	< 500	Esotropia	Nil	95/95	Lateral Rectus	Ortho	Ortho
Patient 11	25	F	0.8	+3.5	400	Exotropia	Myopia	90/90	Medial Rectus	10	Ortho
Patient 12	6	F	0.0	+2	200	Esotropia	Myopia	80/80	Lateral Rectus	2	Ortho
Patient 13	11	M	0.3	+1.5	400	Sensory Exotropia	Cataract/ Amblyopia	90	Medial Rectus	15	8
Patient 14	9	F	0.2	+3.75	700	Exotropia	Amblyopia	90	Medial Rectus	8	Ortho
	
	
F, female; M, male; yr, year; secs, seconds; op, operation; ortho, orthotropia; PD, prism diopters; Log MAR, logarithm of the minimum angle of resolution											

**Table 2:** Descriptive statistics of patients who underwent ocular muscle transplantation in Nigeria between 2019 and 2021


**Variable**	**Mean ± SD**	**Median**	**Confidence interval**	**Range**
Age (yr)	16.6 ± 12.5	16.5	9.4–23.9	1–39
Pre-op deviation (PD)	89.6 ± 9.3	90.0	84.3–95.0	70–110
Pre-op near (PD)	89.3 ± 8.9	90.0	84.1–94.5	70–110
Muscle amount transplanted (mm)	5.7 ± 0.6	6.0	5.4–6.1	4–6
6 months post-op deviation (PD)	6.6 ± 1.8	6.0	2.7–10.5	0–20
	
	
PD, prism diopters; SD, standard deviation; yr, years				

**Table 3:** Comparing preoperative and postoperative distance and near deviation


**Deviation**	**Pre-op distance deviation (PD)**	**Post-op distance deviation (PD)**	**Mean difference (PD)**	***t*** **, ** ***P*** **-value**
Distance deviation	89.6 ± 9.3	6.6 ± 1.8	83.1 ± 13.3	23.313, < 0.001 ${\;}^{*}$
Near deviation	89.3 ± 8.9	6.6 ± 1.8	82.7 ± 12.9	23.983, < 0.001 ${\;}^{*}$
	
	
${\;}^{*}$ Significant at 95%; *t*, *t* test statistic PD, prism diopters				

**Table 4:** Recommended surgical doses tables for large and extra-large strabismus (quoted with permission).^[20]^


**Guidelines for the surgical dosage for transplantation of extraocular muscles**			
**Esotropia (PD)**	**LR resection (mm)**	**MR recession (mm)**	**Transplantation (mm)**
60	8	5.5	3
70	8	6	4
80	8	6.5	5
90	9	6.5	6
**Exotropia (PD)**	**MR resection (PD)**	**LR recession (PD)**	**Transplantation (PD)**
60	6	7	3
70	7	8	4
80	8	9	5
90	8.5	10	6
	
	
LR, lateral rectus; MR, medial rectus; PD, prism diopter; mm, millimeter			

**Table 5:** Recommended expanded surgical tables for extra-large strabismus (Adio, Ezisi, and Nkanga, 2023)


**Esotropia (PD)**	**LR resection (mm)**	**MR recession (mm)**	**Transplantation (mm)**	**Second eye MR recession (mm)**	**Second (mm) eye LR resection (mm)**
70	8	6	4	–	–
80	8	6.5	5	–	–
90	9	6.5	6	–	–
100	9	6.5	6	4	–
110	9	7	7	5	–
120	9	6.5	6	4.5	5.5
130	9	6.5	6	5.5	6.5
**Exotropia (PD)**	**MR resection (mm)**	**LR recession (mm)**	**Transplantation (mm)**	**Second eye LR recession (mm)**	**Second eye MR resection (mm)**
70	7	8	4	–	–
80	8	9	5	–	–
90	8.5	10	6	–	–
100	7	9	6.5	9	–
110	8	10	6.5	10	–
120	8	9	5	7.5	5
130	8	9	5	8	6.5
	
	
LR, lateral rectus; mm, millimeters; MR, medial rectus; PD, prism diopters Best to apply transplant surgery to the less dominant eye or eye with less vision					

**Figure 1:**
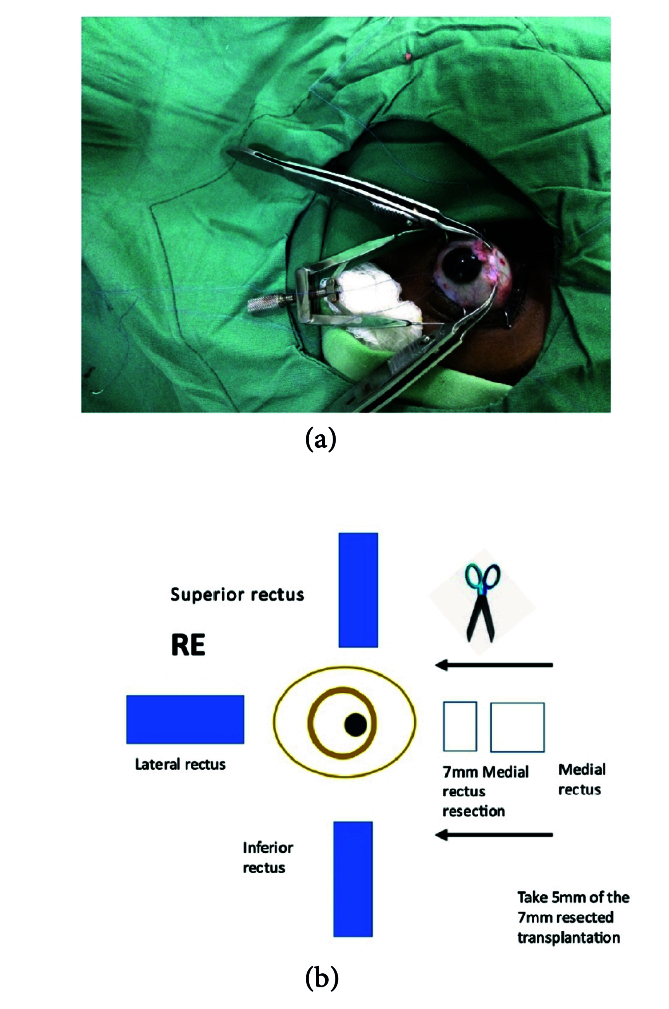
(a) 5 mm of transplantable medial rectus isolated. (b) Illustration of medial rectus to be transplanted.

**Figure 2:**
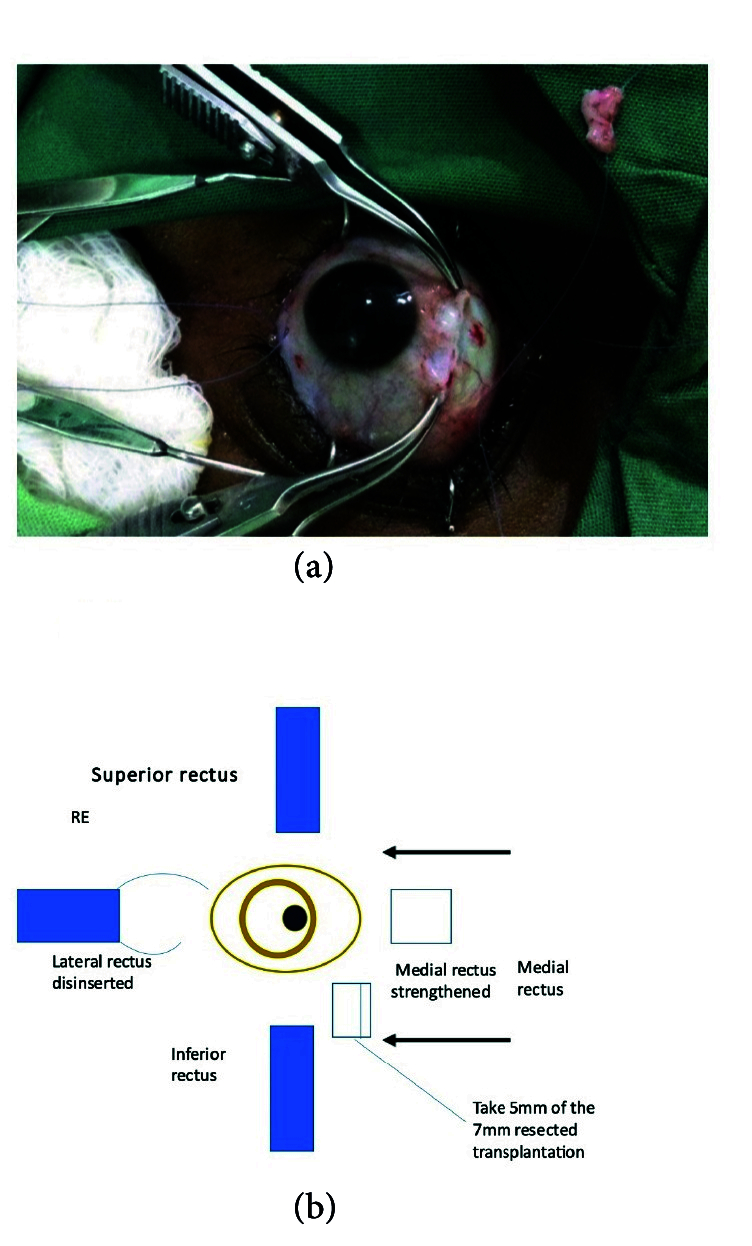
Extraocular muscle to be transplanted with 6-0 double ended. VicrylⓇ    suture with spatulated needle attached to it (on green linen to the top right).

**Figure 3:**
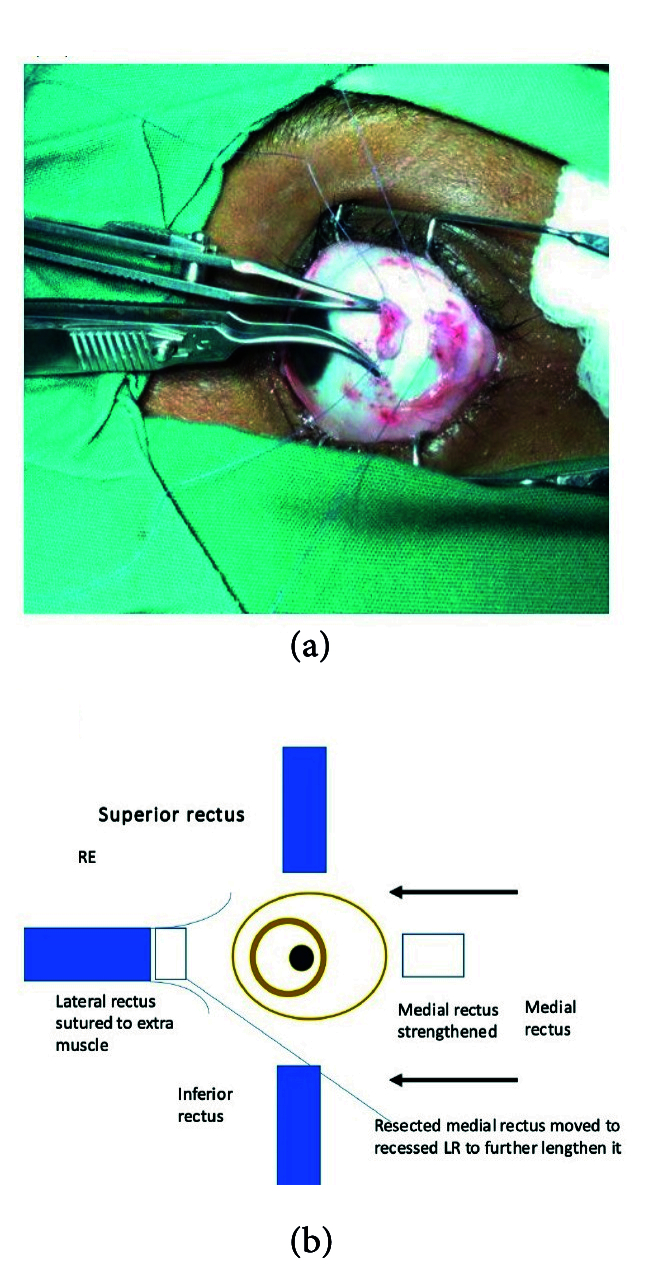
Transplantation of resected medial rectus to the lateral rectus about to take place.

**Figure 4:**
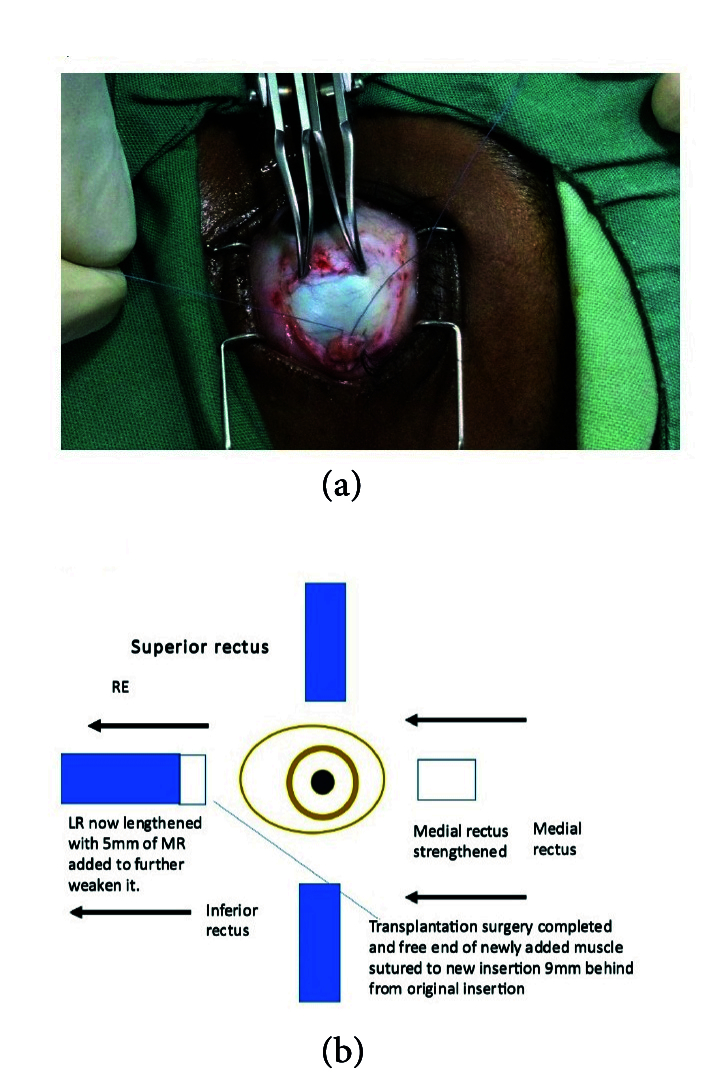
Recession of lateral rectus to 9 mm behind insertion and fixed suture bites to sclera taken after lengthening it.

**Figure 5:**
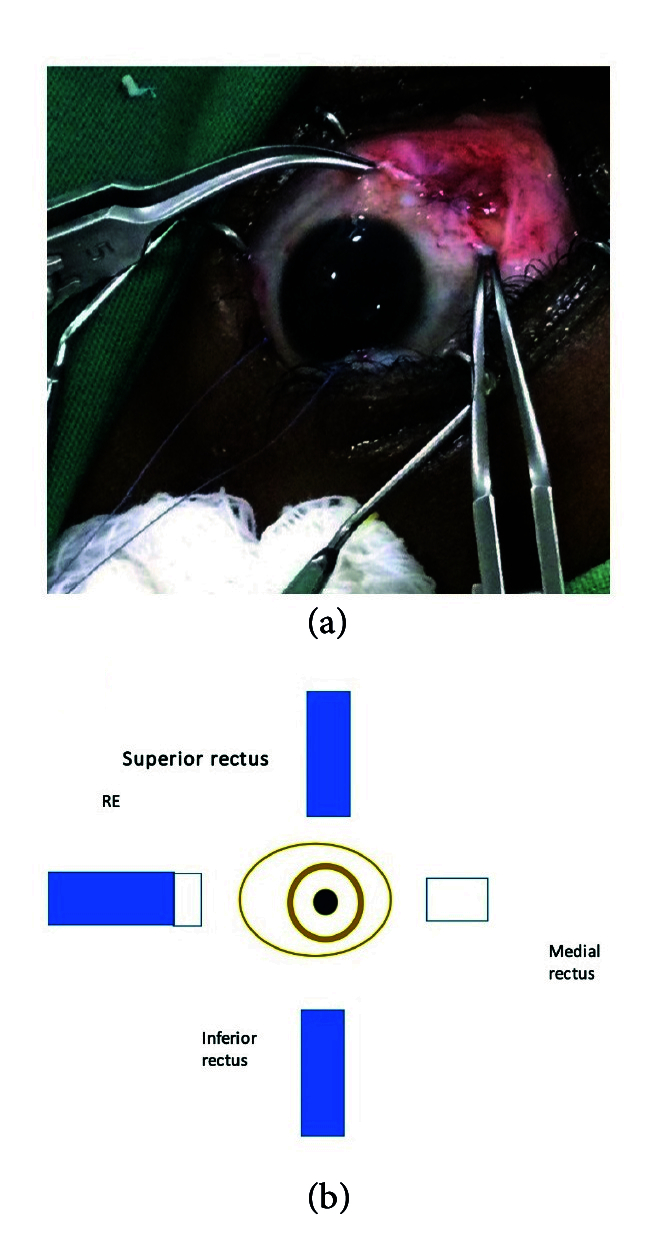
Remaining medial rectus inserted back to original insertion (5.5 mm from limbus) to further strengthen it. Surgery completed.

**Figure 6:**
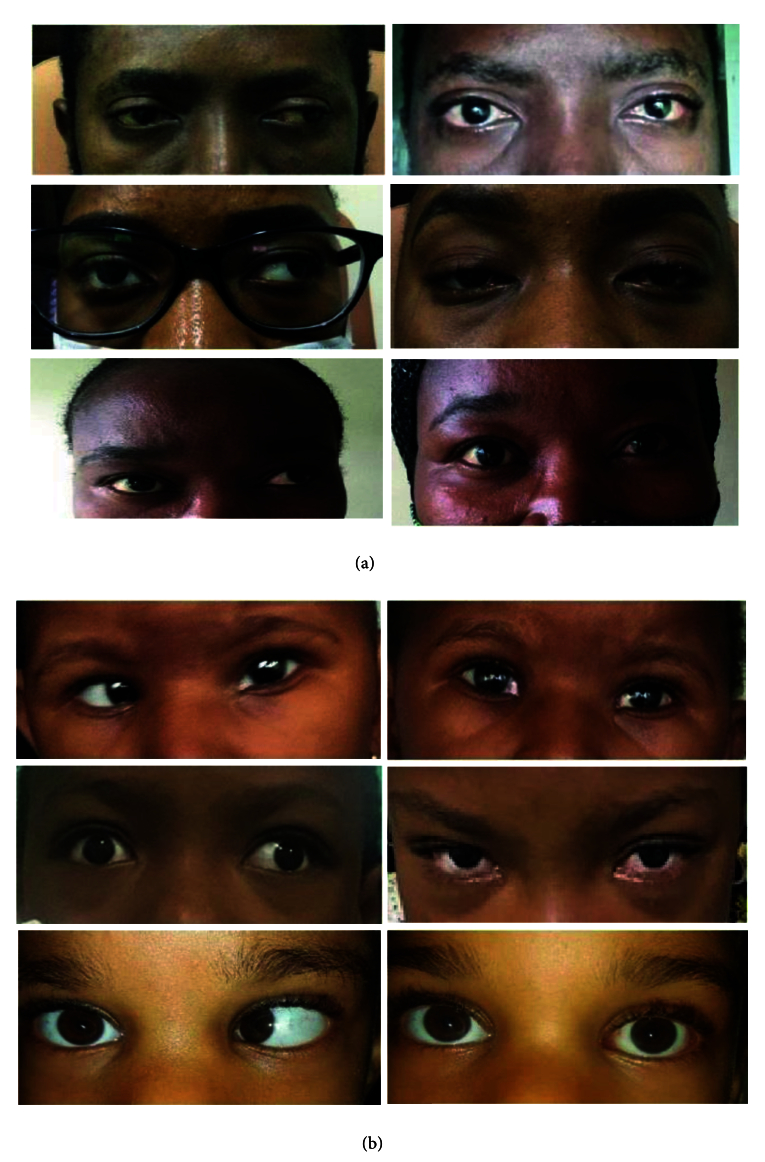
(a) Before and after ocular muscle transplantation surgery pictures of patients in adults with binocular vision and in sensory strabismus. (b) Before and after ocular muscle transplantation pictures in children with large angle strabismus.

### Description of Surgical Technique 

Following the complete orthoptic evaluation, patients with XT undergo transplantation of the MR which is resected off and attached to the LR. In cases of ET, the LR is resected off and attached to the MR. For example, the surgical plan for 90 PD ET consists of performing LR resection of 9 mm and transplanting 6 mm of the resected LR to MR, then recessing the elongated MR by 6.5 mm. The surgical plan for XT of 80 PD comprises performing LR recession of 9 mm associated with MR resection of 8 mm and transplanting 5 mm of MR to LR in the eye that is less dominant with reduced visual acuity. The authors recommend not cauterizing the donating muscle during resection and keeping the transected muscle in sterile saline.^[20]^

Currently, two methods are available.

Method 1:^[20]^ The muscle that is the donating tissue (the MR muscle in this instance) is strengthened by resecting off the piece near the insertion [Figures 1 & 2] according to the particular dosage advised on the extraocular muscle transplant tables^[20]^ [Tables 4 & 5] and reinserting the remaining stump back into the MR's original insertion [Figure 5]. The donor tissue (resected MR) is cleaned and placed in a galley pot with some sterile saline while waiting for the recipient site to be prepared. The recipient muscle (here the LR muscle) is isolated and disinserted; then the donor tissue (from the resected MR) is attached to the disinserted end of the LR using 6-0 Vicryl sutures [Figure 3] according to the tables for muscle transplantation^[20]^ [Tables 4 & 5]. This elongates the LR and the whole segment is recessed to a predetermined distance away from the insertion depending on the initial measurement of the squint [Figure 4]. Following this, the conjunctiva is sutured using an 8-0 Vicryl suture, thus completing the surgery [Figure 5].

Method 2:^[1]^ Essentially, in this method, after isolating the muscle to be recessed (here the LR), using a large muscle hook, the donor muscle tissue is then retrieved (here the fragment of the resected MR) and sutured onto the inserted end of the LR muscle while it is still inserted in sclera. The new donor MR fragment – LR recipient segment (elongated muscle) – is then recessed to a predetermined distance away from the insertion [Figure 5]. The conjunctiva is then closed as is explained in Method 1.

All relevant data such as preoperative and postoperative measurements from day one, week six, and month six after surgery in addition to associated history and clinical ocular examination findings which was taken from patients' folders obtained from the Medical Records Department of the hospitals. Descriptive statistics were checked using simple arithmetic means and proportions. *P*-value of < 0.05 was considered to be statistically significant.

The proposal for this study was reviewed and approved by the Ethics Committee at the University of Port Harcourt Teaching Hospital, Rivers State, Nigeria with the approval number UPTH/ADM/90/S.11/VOL.X/1487. Informed written and verbal consent was received from all participants including the parents/legal guardians of those < 18 years of age. All study participants agreed and signed to have their de-identified pictures shown. The study was in accordance with the revised version of the Declaration of Helsinki.

##  RESULTS

Fourteen patients with extra-large-angle squints underwent ocular muscle transplantation strabismus surgery between 2019 and 2022 in three institutions [Table 1]. The male–female ratio was 0.6:1. The mean age of the patients was 16.6 ± 12.5 years (CI: 9.4–23.9); 50% of the patients were < 18 years. The mean best corrected visual acuity (BCVA) was 0.19 LogMAR. The mean spherical equivalent was +1.18 D. Most of the patients had XT (*n* = 11, 78.6%) with many patients requiring the MR to be transplanted to the LR. Most had no additional ocular pathology (*n* = 8, 57.1%); of the remaining, four (28.6%) had myopia followed by amblyopia secondary to cataract (*n* = 2, %). The mean preoperative deviation was 89.6 ± 9.3 PD (CI: 84.3–95) for distance deviation and 89.3 ± 90 PD (CI: 84.1–94.5) for near deviation. An average of 5.7 ± 0.6 mm (5.4–6.1 mm) of the extraocular muscles were transplanted [Tables 2 & 3].

The postoperative deviation collapsed to 6.6 ± 1.8 PD at six weeks and continued to improve to a mean deviation of 2.5 PD at six months. This improvement was statistically significant both for distance (Student *t*-test 23.313, *P* < 0.001) and for near (Student *t*-test, 23.983, *P* < 0.001).

There were no postoperative abduction or adduction limitations noticed in any of the patients after ductions were performed.

### Comparison of Outcomes of Sensory Strabismus and Strabismus with Good Vision

Four patients had sensory strabismus (*n* = 4, 28.9%). The preoperative deviation of patients with sensory strabismus was 92.5 D. Following surgical correction, this reduced to 5.9 D of deviation. The remaining 10 patients in our series had good vision in both eyes (*n* = 10, 71.9%). The preoperative deviation of these patients with good vision was 88.5 D. Following surgical correction, this collapsed to 1.0 D of deviation.

### Comparison of Outcomes Between Pediatric and Adult Extra-large Strabismus Following Extraocular Muscle Surgery

Surgical outcomes for patients who were 18 years old and younger was compared with the outcomes of patients older than 18 years.

Of the seven pediatric patients, the male/female ratio was 1.5:2 and the average age was 5.6 ± 3.6 years. The mean preoperative deviation was 89.3 PD. This collapsed to 1.4 PD six months after the surgery. In comparison, of the seven patients that were older than 18 years, the male-to-female ratio was 1:2.5 with an average age of 27.7 ± 6.5 years. The mean preoperative deviation for this group was 90 PD which decreased to 3.3 PD six months after the surgery.

##  DISCUSSION

Extra-large-angle squints mainly develop due to late presentation for treatment. Reasons given for late presentation include ignorance, poverty, and lack of available skilled specialists. In this study, the average age of presentation for those with extra-large-angle deviations was 16.6 years which was much older than those found in a study by Azonobi et al.^[21]^ Azonobi et al, whose study was carried out amongst school children in Nigeria, found the average age of presentation to be 9.5 ± 6 years.^[21]^ However, the findings from our study was more consistent with older age at presentation in a Saudi Arabian study by Curtis et al.^[22]^ This disparity is probably due to the fact that the children who were examined in school were minors and uninformed about their ocular condition and were also not able to make informed decisions by themselves to take up surgical correction. However, the age of presentation for our patients [Table 1] was much younger than an Indian series of 22 patients presented by Jethani et al (32.21 ± 13.1 years).^[23]^ The average size of the deviation of 92.4 PD in the Indian study by Jethani et al^[23]^ is in keeping with our study where we found approximately 90 PD in our patients. However, some other studies found that 60 PD was the average size of the squint among those who presented.^[24]^

Since the discovery of effective surgical therapies for the treatment of squint, possible surgical options have evolved for treating extra-large squints that exceed 70 PD if the deviation is comitant which include very large recess–resect surgery, the hang back method,^[25]^ and the use of silicone expanders.^[26]^ If the deviation is incomitant, transpositions^[27]^ and periosteal muscle anchoring surgeries are performed.^[28,29]^

However, these methods have demonstrated significant limitations as most of these techniques include the use of synthetic materials which may lead to excessive inflammatory reactions. In addition, in some cases the muscle to be adjusted was shown to reinsert in inaccurate positions instead of the intended spot as in the hang back method. The possibility also exists that the sutures could break.

Once a squint is > 70 PD, traditional squint muscle surgery may not help due to the limitation of the length of individual extraocular muscles (the average amount of correction per millimeter of muscle is responsible for this).^[30]^

The development of the muscle transplantation technique reduces surgical morbidity to one eye while leaving the other eye in its virgin state for possible future surgery. This development has made extra-large-angle squints amenable to correction using much fewer muscles and with less risk by using the patient's own muscle rather than synthetic materials.^[23]^

Repeated squint surgeries lead to scarring at the surgical site and formation of adhesions, making future surgeries difficult.^[31,32]^ Therefore, there is a need to achieve full correction with the use of the minimal number of horizontal muscles. This is an advantage of muscle transplantation surgery that it strengthens the moment arm of the muscle by adding the donated resected muscle to further lengthen the muscle to be weakened. This means that one of the horizontal rectus muscle is elongated. This elongation is achieved by resecting a portion of the opposite horizontal rectus muscle and transplanting it onto the muscle to be elongated. Thus the donor muscle becomes shorter and the recipient muscle becomes longer. The elongated muscle is further recessed away from its original point of insertion. The donor muscle, which is now shorter in length, is reattached to its original point of insertion. This way, extremely large-angle squints can be corrected even if > 100 PD.^[19,23,33]^ On average, a millimeter of transplanted muscle gives up to 4–5 mm additional effect, which is compatible with our experience.^[19]^

To guide this appropriately, in a measurable and consistent way, surgical dose tables have been developed by the current authors^[31,34]^ [Table 5] and have proven useful in such instances when the size of the squint is larger than in previously available standard squint charts.^[23]^ Therefore, unilateral muscle transplantation combined with the recession–resection procedure could be a useful alternative for the management of extra-large-angle ET or XT.^[15,34,35]^ We have developed an expanded surgical table incorporating our experience and the outcomes of our surgeries which can be used as a guide and recommendation for treatment of similar extra-large-angle squints that present (mostly in developing countries) for which there are presently no surgical dosage tables [Table 5]. This table has been found useful and it is recommended particularly when one eye is dominant. Ocular muscle transplantation can be offered to the patient for the less dominant eye while a recess–resect procedure can be done in the other eye for the remaining size of deviation [Table 5].

Concerning the cosmetic treatment of sensory strabismus as explained by Tibrewal et al,^[34]^ instead of having to operate on both eyes as the standard tables suggest (which may sometimes be not acceptable), muscle transplantation can be offered which limits the surgery to just one eye. However, if very large squints exist (e.g., > 100 PD), the other eye can also be operated upon either by selecting to operate on one muscle or both horizontal muscles in the other eye (recess–resect surgery) as outlined in our new expanded surgical dosage table [Table 5].

In our study, both of those patients whose surgery was either purely cosmetic (28.6%; six months postoperative deviation of 5.8 PD) or for standard treatment (71.4%; six months postoperative deviation of 1 PD) had good outcomes following this technique with slightly better outcomes among those with binocular vision. Other studies have also proven the effectiveness of this landmark procedure.^[23,30,34,35]^

The transplanted muscle survives and remains viable; however, it undergoes changes, that is, necrosis and obliteration of its capillaries with gradual fibrous replacement of muscle fibers by 28 days. It does not incite as much reaction from the recipient muscle or sclera. The tensile strength correlates very well to repaired muscle wounds with the union of transplanted muscle to sclera and recipient muscle which becomes very good after 14 days.^[36]^

We used 6-0 double-ended spatulated Vicryl in all our patients with quite good outcomes but others have advocated the use of 6-0 Prolene since it may not biodegrade as fast.^[23]^ However in our series, it was quite adequate to use Vicryl suture 6-0 to keep the muscles intact while fusing together with sclera.

Documented complications associated with this procedure may include lost muscle, over-corrections, new deviations, and infection.^[37]^ Although complications also happen in other surgical procedures, in our study, these were not noticed in any of our patients. This may be because our report is on a relatively small number of patients. Some studies with slightly larger series have also reported minimal complications following this procedure.^[23,30]^

Ocular muscle transplantation can be used in combination with other methods of correcting squint, for example, in treatment of heavy eye syndrome where it can be used in combination with loop myopexy.^[19]^ Other specialists have reported good results for residual and recurrent strabismus as we had in one of the patients in our series.^[38]^

This technique can also be used in both eyes, especially when the binocular vision is present, achieving quite good results.^[1]^ The tables we developed [Table 5] can also be used quite comfortably in this situation.

Some specialists have tried using the oblique muscles instead of the medial or lateral recti,^[39]^ while other specialists studied alternatives aside from utilizing real muscle such as fascia lata especially in patients with complex ocular motility disorders like thyroid eye disease^[40]^ and congenital innervation disorders for which conventional strabismus surgery is insufficient. Surgery with tendon elongation allows correction of larger angles and is able to maintain a sufficient arc of contact for rectus muscles. Bovine pericardium called TutopatchⓇ for tendon elongation has been used in previous studies.^[41]^

In summary, the indications for performing muscle transplantation include the presence of extra-large squints ( > 70 PD), previous squint surgery that did not fully correct the deviation, when the patient wants only the diseased eye that is amblyopic or blind be operated, presence of paralytic squint, and in the event of LR palsy with good abduction. Muscle transplantation can be carried in both eyes for extra-large deviations if vision is the same or near equal in both eyes. Our new expanded surgical dosage table provides a recommended surgical plan for up to 130 PD of deviation. These surgical dosages were developed by the current authors from the experiences gathered from treating the patients in our study [Table 5].

The advantages of the procedure are that it is physiologic, and no foreign elements are used. Nothing is wasted, as previously resected muscle is transplanted. In addition, surgery on dominant eyes can be avoided especially if the deviation is < 100 PD with better preservation of ductions. It also has a simple learning curve and it is very likely that the patient will only need one procedure to correct the full deviation.

Muscle transplantation is a safe option for extra-large-angle squint. The surgical results are stable in the long term even in children and hence a viable option to treat extra-large-angle squints in both adults and children.The limitation of our study was the small number of patients who underwent this procedure.

##  Financial Support and Sponsorship

None.

##  Conflicts of Interest

None.
